# Dispersion-Controlled Excited-State Dynamics in Azobenzene Photoisomerization

**DOI:** 10.1021/jacs.5c16915

**Published:** 2025-12-30

**Authors:** Torben Sassmannshausen, Nils Oberhof, Marcel A. Strauss, Chavdar Slavov, Hermann A. Wegner, Andreas Dreuw, Josef Wachtveitl

**Affiliations:** † Institute of Physical and Theoretical Chemistry, 9173 Goethe University, Frankfurt 60438, Germany; ‡ Interdisciplinary Center for Scientific Computing, 9144 Heidelberg University, Heidelberg 69120, Germany; § Institute of Organic Chemistry, Justus Liebig University Giessen, Giessen, 35392, Germany, and Center of Material Research (LaMa/ZfM), Justus Liebig University Giessen, Giessen 35392, Germany; ∥ Department of Chemistry, 7831 University of South Florida, Tampa, Florida 33620, United States

## Abstract

Weak interactions, like London dispersion forces, are cumulative in nature and have been thought to be essential for only the structure and stability of large molecular systems. Only recently has their relevance for chemical reactivity been recognized. Until today, their role in photoreactions and subsequent ultrafast excited-state processes has remained elusive. Here, we show the impact of London dispersion on the excited-state behavior and the outcome of the photoreaction of the iconic photoswitch azobenzene as a representative example. Increased dispersion interactions between substituents decisively prolong the excited-state lifetimes by preventing direct passage through the conical intersection. This significantly alters the dynamics of the *Z* to *E* photoisomerization. We expect our findings to lead to increased research interest in such “dispersion-controlled excited-state dynamics” relevant for the steering of ultrafast processes.

## Graphical abstract

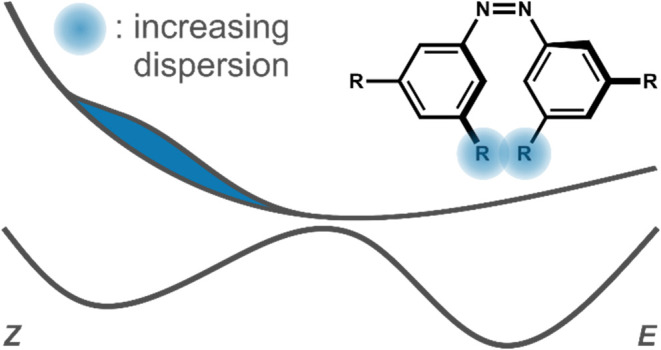

## Introduction

Molecular interactions are fundamental to the structure and behavior of matter, governing the formation and stability of molecules and materials. These interactions encompass a wide range of different strengths, from strong covalent bonds to weak noncovalent interactions. While the importance of covalent and ionic bonds is well-recognized, weaker forces such as hydrogen bonds, van der Waals forces, and particularly dispersion forces have traditionally been considered secondary in the hierarchy of molecular interactions. However, recent advances in experimental and computational methods have illuminated the subtle yet decisive roles these weaker interactions can play in molecular dynamics and structural stability.^1–4^

Among the noncovalent interactions, dispersion forces—especially London dispersion forces—are crucial in stabilizing molecular complexes and influencing their dynamic behavior.^2,4^ These forces, although weak individually, collectively contribute significantly to the overall interaction energy landscape, in particular, of large molecular systems. Recent insights have revealed their profound impact on molecular conformations and reaction pathways, especially in complex chemical and biological systems, challenging the traditional narrative that considered them negligible.

The impact of dispersion forces and, thus, dispersion-controlled dynamics is evident across a plethora of fields. In material science, they facilitate the design of smart polymers^5^ and adaptive surfaces^6^ by allowing controlled manipulation of material properties at the molecular level. Supramolecular chemistry can leverage these forces for enhanced assembly,^7^ stability of host−guest complexes,^8^ and molecular architectures.^9^ Also, in single-molecule junctions, London dispersion interactions have been shown to be operating.^10^ In photophysics and photochemistry, dispersion forces play a pivotal role in light-driven processes,^11–15^ informing the development of solar energy technologies^16,17^ and light-responsive materials.^18^ Moreover, in biochemistry and structural biology, they influence protein folding,^19^ DNA stability,^20^ and the function of chromophores in proteins,^21^ while in pharmaceutical chemistry, they control drug–receptor interactions^22^ and determine the development of photoresponsive therapeutics.^23^ In the realm of nanotechnology, understanding dispersion forces leads to innovative designs of nanoscale devices,^24^ such as sensors.^25^

Herein, we introduce the term “dispersion-controlled dynamics” to describe scenarios in which dispersion forces decisively influence the movement and transformation of molecules. Unlike stronger interactions, dispersion forces are uniquely capable of subtly directing the dynamic behavior of molecular systems without significantly altering their energetic frameworks. This concept highlights the role of dispersion forces not merely as passive stabilizers but as active participants in shaping molecular events, ranging from reaction kinetics to conformational changes.

To unravel the intricate interplay of dispersion forces and molecular motions and to demonstrate how even ultrafast excited-state dynamics are determined by dispersion forces, we explore the photochemistry of azobenzene—the prototypical molecular switch widely used and exploited due to its unprecedented dynamic photoisomerization capabilities, as our model system. Our multifaceted approach integrates organic synthesis, time-resolved spectroscopy with excited-state dynamics simulations, and reveals dispersion to indeed decisively modulate the torsional and inversion motions of azobenzenes, directly impacting the coupling between the S_1_ and S_0_ states at the conical intersection (CI).

This proof-of-principle study introduces “dispersion-controlled excited-state dynamics” as a foundational framework that prompts a re-evaluation of the pervasive yet often underappreciated role of dispersion forces in molecular reactivity and, in particular, excited-state dynamics. Through this framework, we highlight the profound implications of dispersion, offering a gateway to novel applications and theoretical advancements across diverse scientific domains.

## Results and Discussion

### Lifetimes and Populations

The investigated azobenzene derivatives (Figure 1) differ only in the size of their *meta*-substituents. The meta positions are ideal sites for modulating dispersion interactions without altering the electronic properties of the azobenzene.^26–29^ With increasing substituent size, the main ππ* bands of the (*E*)-azobenzene derivatives undergo slight red shifts. This can be explained by the inductive effect of the substituents and is also observed in other alkyl-substituted azobenzenes.^30^ All derivatives can be photoisomerized to the (*Z*)-form (Figure 1 inset) and show similar photostationary state (PSS) spectra (Figures 1 and S1).

**Figure 1:**
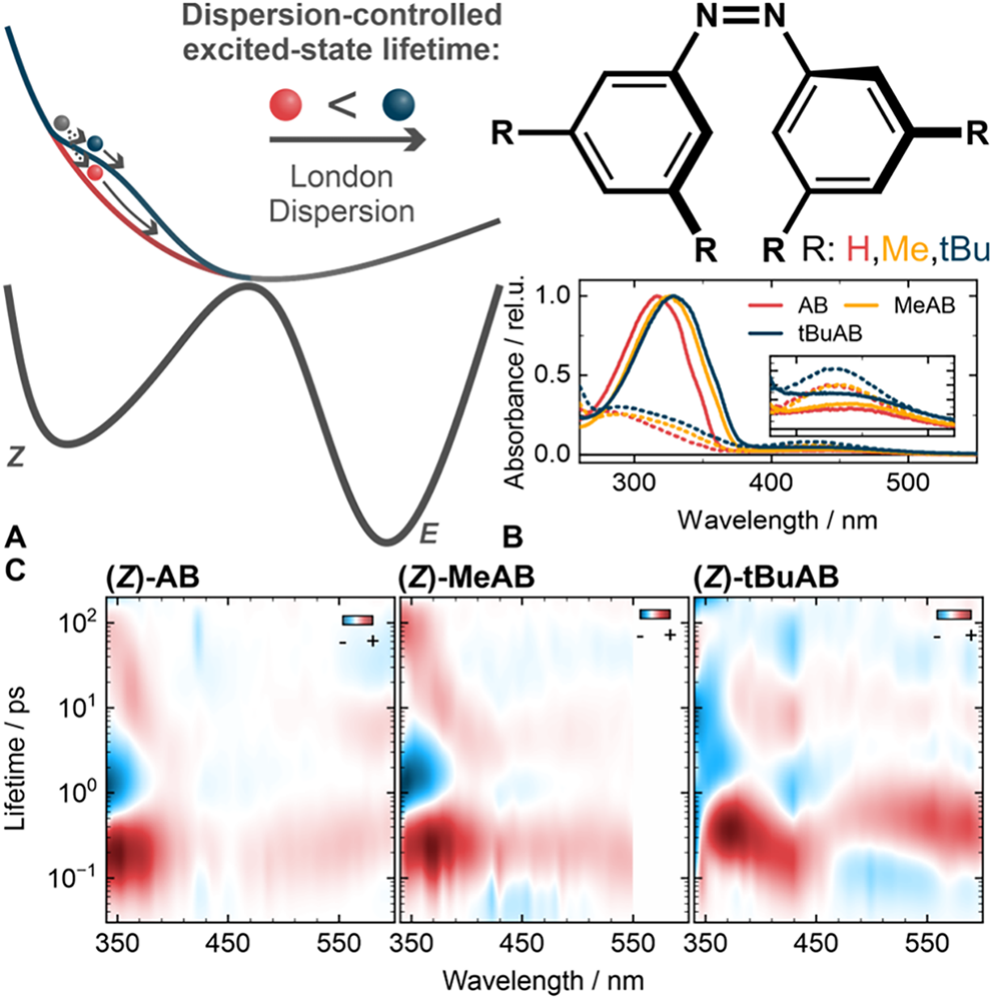
(A) Influence of the London dispersion on the excited-state trajectory of azobenzene is shown in a schematic figure. (B) Scheme of all three azobenzene derivatives investigated in this work, as well as the absorbance spectra (solid lines) and photostationary state data (PSS, dashed lines) in MeCN. (C) Lifetime density analysis of the transient absorption data for (*Z*)-AB, (*Z*)-MeAB, and (*Z*)-*t*BuAB. The positive components describe a rising negative or a decaying positive signal. The negative components depict a decaying negative or rising positive signal. All samples were excited by a 425 nm laser pulse.

To determine the impact of London dispersion forces on the first excited state of the (*Z*)-AB derivatives, the dynamics of (*Z*)-AB were studied in detail using both time-resolved spectroscopy^31–35^ and theoretical approaches.^36–43^ Transient absorption (TA) measurements were conducted in acetonitrile (MeCN) and *n*-octane.

In brief, the TA spectra show two excited-state absorption (ESA) bands around 350 nm and above 470 nm, which both decay with a lifetime of 180 fs ( Figures S2­(A–D) and[Fig fig1]C). The negative ground state bleach signal is located between 420 and 470 nm. The (*Z*) → (*E*) isomerization is detected by the residual ground state bleach signal at 1.8 ns and the newly formed (*E*)-azobenzene photoproduct absorption signal at 350 nm (Figure S2).

The lifetime density maps (LDM)^44^ obtained from the transient absorption data sets show four main contributions (Figure 1C). The first two prominent features at 350 and 550 nm are centered at a 180 fs lifetime and describe the main decay of the S_1_ state.^32^ However, a small population carries out a diffuse motion on the excited-state surface and returns later to the ground state via internal conversion.^32^ This is visible as a small positive density around 400 nm and 2 ps. After the transition to S_0_, the hot ground state isomers undergo vibrational cooling^31^ on a time scale of around 15 ps^32^ (Figures S2 and[Fig fig1]C).

While the general spectral features are very similar for the derivatives (*Z*)-MeAB and (*Z*)-*t*BuAB, the overall dynamic is slowed down, and the corresponding lifetimes are longer (Figure 1C). In (*Z*)-MeAB, the main S_1_ decay occurs with a longer lifetime of 240 fs (Figure 1C). Similarly, the diffusive motion is also slowed, which is visible in the slightly delayed positive feature in the LDM around 400 nm and 2 ps (Figure 1C). However, the cooling of the hot ground state is in the range of 10−20 ps and very similar to that of the parent (*Z*)-AB.

For (*Z*)-*t*BuAB, the S_1_ decay is further slowed with a longer lifetime of 400 fs (Figure 1C). Additionally, the contribution describing the S_1_ decay is governed by a blue-shift, which is present neither in (*Z*)-AB nor in (*Z*)-MeAB. This indicates generally altered excited-state dynamics. In (*Z*)-*t*BuAB, also the lifetime of the diffusive excited-state motion is further prolonged (≈3 ps), and in contrast to (*Z*)-MeAB, the cooling process now takes around 50 ps.

The difference between the excited-state decay processes of the differently substituted (*Z*)-AB derivatives and the clear evidence of a shift toward longer lifetimes demonstrate the dependence of the photoinduced dynamics on the specific substituents. Since measurements in octane show very similar distributions (Figure S3) and the short lifetimes of azobenzene have been shown to be independent of viscosity,^35^ the observed deceleration in the substituted (*Z*)-AB derivatives does not originate from solvent or viscosity effects. Importantly, London dispersion interactions generally do not cancel out in solution^45^ and show only a slight viscosity dependence in alkane solvents.^46^ Therefore, the different London dispersion interactions between the substituents can be considered the main cause of the observed differences in the excited-state dynamics.

Further insights into the role of London dispersion for the excited-state dynamics of these azobenzenes are gained through excited-state ab initio molecular dynamics simulations using OM2/MRCI fewest switches surface hopping (FSSH) dynamics with and without the D3­(BJ) dispersion correction (for details, see Computational Methods section). The populations of the ground (S_0_) and excited (S_1_) states for the first 500 fs, calculated without dispersion interaction (Figure 2A–C), show that the time needed for a 50% population decay to the ground state (indicated by dashed lines) is very similar for all three compounds. Hence, the increasing inertia with growing substituent size can be excluded as the cause for the increasing lifetime observed in the experiment. Only the inclusion of dispersion effects in the excited-state dynamics simulations leads to longer S_1_ population lifetimes with increasing substituent size (Figure 2D–F). This effect is most pronounced in the case of (*Z*)-*t*BuAB. For comparison with the simulations, global target analysis was used to obtain corresponding experimental state populations (Figure 2G–I), which agree very well with those of the theoretical populations, including dispersion interactions (Figure 2D–F) and manifest the concept of ”dispersion-controlled excited-state dynamics”.

**Figure 2:**
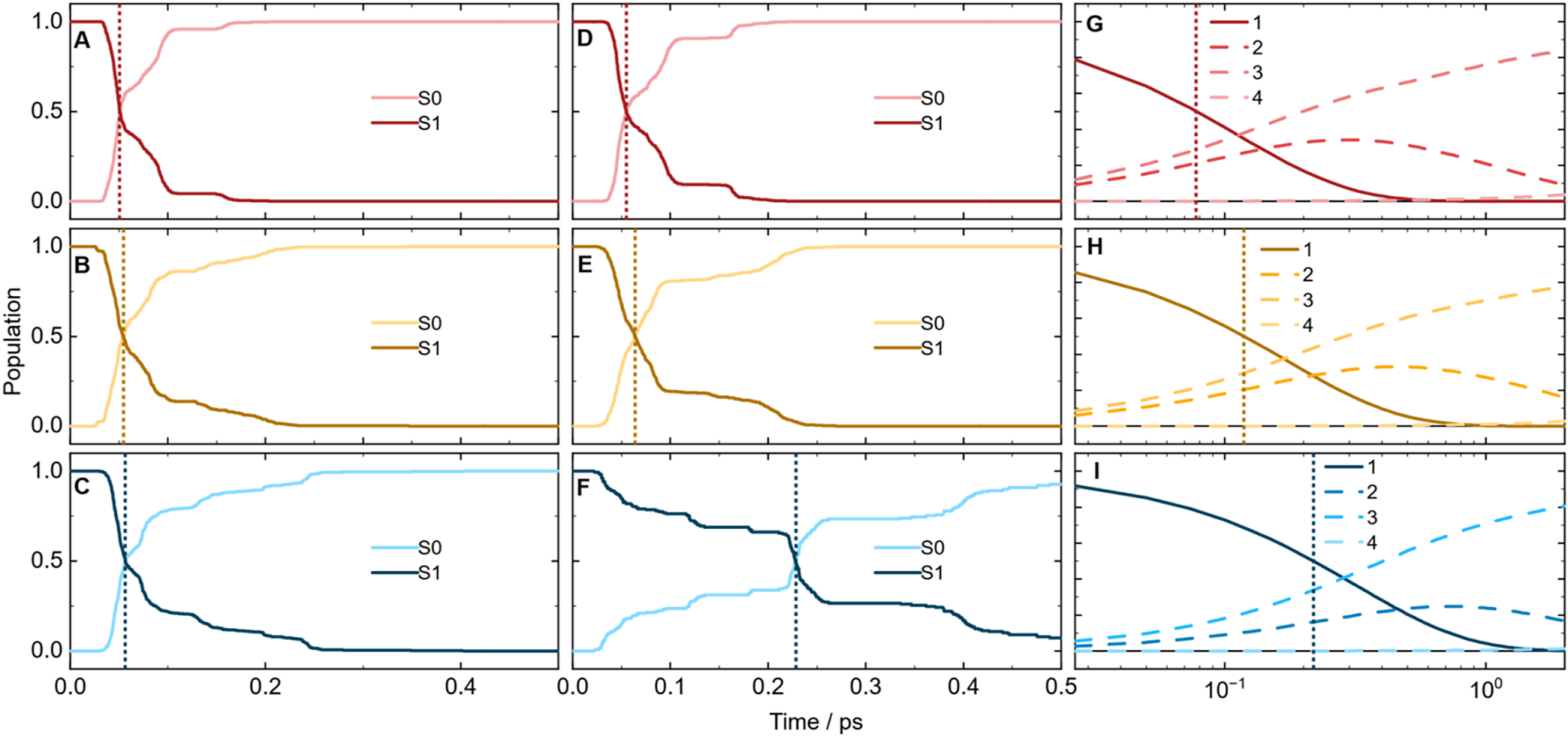
(A–C) Calculated population curves without dispersion correction for (*Z*)-AB, (*Z*)-MeAB, and (*Z*)-*t*BuAB, respectively. (D–F) The same populations are calculated, including the dispersion interaction. (G–I) Calculated populations were obtained from a global target analysis of the transient absorption data. The main S_1_ decay is represented by population **1**, while population **2** is the small remaining S_1_ population. The third population (**3**) shows the cooling dynamics, while the fourth (**4**) represents the (*E*)-isomer.

### Induced Coherent Oscillations

The transient absorption data of (*Z*)-*t*BuAB shows a strong oscillatory modulation on the sub-ps time scale in both solvents (MeCN: Figure 3A; octane: Figure S4­(A)). The oscillating signal is present in both ESA bands, lasts until 1 ps, and is due to additional molecular motions on the potential energy surface. Fourier transformation of the residual (fit of the exponential dynamics minus experimental data) yields the frequency spectrum of the oscillatory motion (MeCN: Figure 3B; octane:Figure S4­(B)). The spectrum shows a dominant 40 cm^−1^ mode and a second component with a frequency of 120 cm^−1^ with a smaller amplitude. For (*Z*)-MeAB, these signal modulations are hardly visible and barely quantifiable, while in the case of (*Z*)-AB, these oscillations are not observed at all. The amplitude of these oscillations increases with substituent size, indicating a direct relationship between them. To check whether dispersion forces also influence these oscillatory motions, the vibrational normal modes of (*Z*)-*t*BuAB were calculated with and without dispersion correction (OM2/MRCI ± D3­(BJ)).

**Figure 3:**
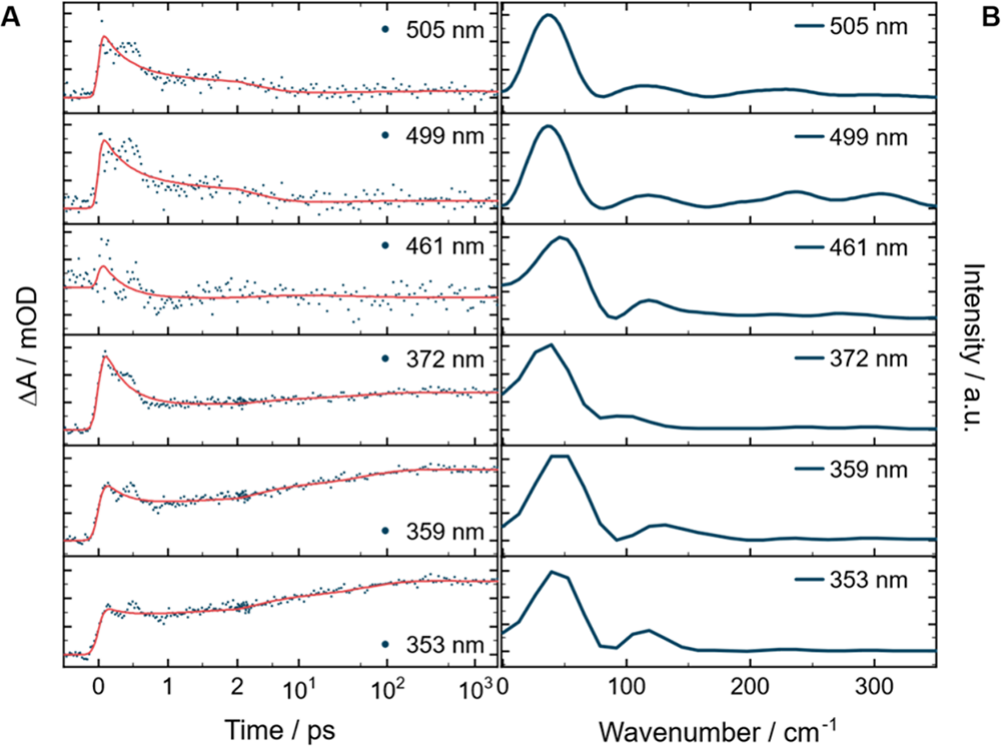
Single transients at selected wavelengths of (*Z*)-*t*BuAB in MeCN (A) and the corresponding frequency spectra obtained from Fourier analysis of the residuals (B).

Indeed, three low frequency normal modes were found to be sensitive to the dispersion correction (Figure S5): (i) an asymmetric CNN and NNC bending mode with a frequency of 51.51 cm^−1^, (ii) a CNNC twisting mode with a frequency of 52.56 cm^−1^, and (iii) a combination mode of CNNC twisting and *t*Bu-rotation with a frequency of 64.55 cm^−1^. These three modes closely resemble the induced excited-state dynamics, since the CNN and NNC angles, as well as the CNNC dihedral angle, are known to be the critical coordinates for reaching the conical intersection on the S_1_ surface during the (*Z*) to (*E*) isomerization.^47^ Since exactly those vibrations resembling this motion have been shown to be sensitive to the London dispersion forces, it is clear that also the (*Z*) to (*E*) isomerization pathway is influenced by the strength of the dispersion interactions. Finally, since the dispersion interactions between the substituents increase in the order (*Z*)-AB, (*Z*)-MeAB, and (*Z*)-*t*BuAB, it is clear that the occurrence of the oscillatory motion along the isomerization pathways in this order is invoked by London dispersion.

### Reactive Coordinates

For (Z)-*t*BuAB, the time evolution of both the CNN angles and the CNNC torsion was extracted from the excited-state surface hopping molecular dynamics. For investigating the differences between fast and slow isomerization processes, the trajectories were separated into different time intervals. The latter were chosen based on the time distribution of the hopping events back to the ground state (see Figure S6). For (Z)-AB and (Z)-MeAB, a population of up to 150 fs and a population of above 150 fs have been defined. In the case of (Z)-*t*BuAB, three relevant time intervals up to 150 fs, between 150 and 300 fs, and above 300 fs have been identified. For all three AB derivatives (see Figures S7−S9), the populations show very similar patterns up to 150 fs (Figure 4A, C, and E). The CNNC angle changes to −90° within 50 fs, and at the same time, a large asymmetry (large ΔCNN values) in the CNN and NNC angles is observed, which represents the CI geometry^47^ and leads to a large number of hopping events before 150 fs. However, the number of these early hopping events decreases with increasing substituent size (Figure S6). For (Z)-AB, 90.9% of the populations hop within the first 150 fs, while for (Z)-MeAB and (Z)-*t*BuAB, this value drops to 83.4% and 31.2%, respectively. The trajectories of all three molecules that undergo S_1_ to S_0_ hopping only during the later time intervals also show common features. Again, the CNNC angle reaches −90° quickly, but starts to oscillate afterward. The asymmetry in the CNN and NNC angles observed for (*Z*)-AB and (*Z*)-MeAB (Figure 4B,D) is smaller for (*Z*)-*t*BuAB (Figure 4F,G). Hopping to the ground state surface and completion of the isomerization thus do not seem possible when the dihedral angle quickly reaches −90° for the first time. Since these molecules simply miss the CI, hopping takes place only when both conditions are fulfilled. Due to the increased London dispersion forces, the asymmetry of the CNN and the NNC angles decreases with increasing substituent size and, concomitantly, the number of later hopping events increases from (*Z*)-AB to (*Z*)-MeAB and (*Z*)-*t*BuAB (see Figure S6).

**Figure 4:**
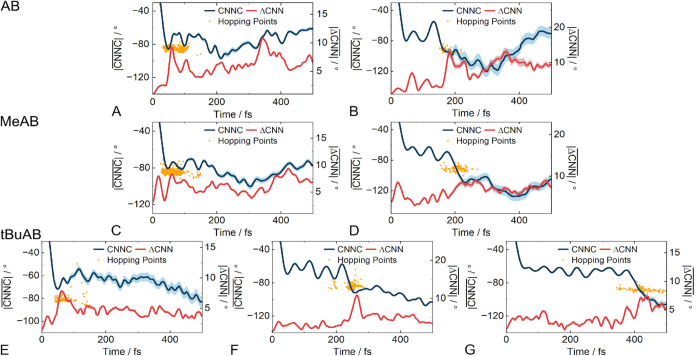
Evolution of the averages of the CNNC dihedral and the ΔCNN angle for the trajectories with the hopping time interval <150 fs for (*Z*)-AB (A), (*Z*)-MeAB (C), and (*Z*)-*t*BuAB (E). Further, the evolution of the trajectories that hop after 150 fs ((*Z*)-AB (B) and (*Z*)-MeAB (D)). For (*Z*)-*t*BuAB, additional time intervals are defined as 150 fs–300 fs (F) and above 300 fs (G). All hopping events are indicated as yellow dots (on the CNNC scale), and the mean error is displayed as a colored area around the line.

In summary, the stronger London interactions, introduced through enlarged substituents, lead to different dispersion-controlled dynamics on the corresponding excited-state potential energy surfaces. The differences manifest themselves in occurring coherent oscillations and increased excited-state lifetimes of (*Z*)-*t*BuAB. To the best of our knowledge, this is the first direct proof of London dispersion influencing the excited-state dynamics of a fundamentally important photoswitch like azobenzene.

## Conclusion

In this work, the direct influence of London dispersion forces on an ultrafast photoreaction is demonstrated for the first time using the iconic (*Z*) to (*E*) isomerization of azobenzene as a fundamentally relevant example. Indeed, the observed “dispersion-controlled excited-state dynamics” are altered, and the excited-state lifetimes are prolonged, with increasing substituent size and increasing London dispersion forces within the azobenzene derivatives. By employing time-resolved spectroscopy in combination with excited-state dynamics, the delayed decay of the excited state could be traced back to the central torsion and inversion coordinates, which are crucial for reaching the S_1_/S_0_ conical intersection mediating the (*Z*) to (*E*) isomerization of azobenzenes. For (*Z*)-*t*BuAB, the increased London dispersion forces lead to increased symmetry between the CNN and NNC angles, i.e., the inversion coordinate, and an enhancement of the torsion coordinate, bringing these simultaneously required motions out of phase. This prevents the direct realization of the CI geometry and causes the delayed excited-state decay and decelerated dynamics. It is apparent that this out-of-phase behavior becomes more and more dominant as the London dispersion interactions between the substituents in the ABs become larger. In fact, the excited-state dynamics are thus controlled by the dispersion interactions, or in other words, they are “dispersion-controlled excited-state dynamics”. This work will inspire further research into steering fundamental photochemical processes by tailored dispersion interactions.

## Experimental Section

### Photoswitch Synthesis

The synthesis of azobenzene (AB), 3,3′,5,5′-tetrametheylazobenzene (MeAB), and 3,3′,5,5′-tetra-*tert*-butylazobenzene (tBuAB) has been according to the literature.^11^

### Steady State Spectroscopy

All experiments were performed in acetonitrile or *n*-octane. For steady state absorbance experiments, a standard spectrophotometer (Specord 600, Analytik Jena) was used, and the OD was set to 1. Two LEDs (Thorlabs M325L5 and Thorlabs M420L2) were used as a light source in the illumination experiments. For better comparability, all absorbance data sets were normalized to the maximum absorbance around 320 nm of the dark spectrum.

### Quantum Yield

All quantum yields were calculated by measuring the absorbance change at 440 nm by a photospectrometer (Jasco, V-650), while the sample was irradiated with a 420 nm LED (Thorlabs M420L2). The absorption change was monitored every 0.1 s for 4 min. For all experiments, 10 mm × 10 mm cuvettes, a sample volume of 3 mL, and an OD of 1 were used. During the measurement, the sample was stirred and held at a constant temperature of 293 K. The illumination was done by focusing the LED into an optical fiber, guiding the light directly above the cuvette, thus measuring the quantum yield in the high OD regime. The excitation intensity was determined by an actinometer.^48^ and the quantum yield was determined as previously described in the literature.^49^ To account for the rising absorption of the (*E*)-AB derivative, we used the difference in the extinction coefficient of the (*E*)- and the (*Z*)-isomers.^50^ For all compounds, we determined the quantum yield for three different power values.

### Ultrafast Spectroscopy

Transient absorption spectroscopy in the visible spectral range was performed on a home-built pump−probe setup as described in detail previously.^49^ In brief, the femtosecond laser system consists of a Ti:sapphire amplifier system (Clark, MXR-CPA-iSeries), which generates the fundamental laser pulses (1 mJ, 775 nm, 130 fs, 1 kHz). The pump pulse was generated by a home-built two-stage NOPA with a prism compressor between them for pulse compression. Afterward, the NOPA pulse is used as a seed for generating the SFG pulse at 425 nm by mixing with the fundamental. A white light continuum was used to probe the dynamics. The continuum was generated by focusing the fundamental into the CaF2 window (3 mm). The probe pulse was focused on the sample and afterward analyzed in a spectrograph (AMKO Multimode) containing a grating with 600 grooves/mm blazed at 300 nm and a photodiode array. The reference beam was directly guided to a second spectrograph with identical specifications. The sample was prepared in a 1 mm quartz cuvette that was moved continuously to avoid photodegradation. The OD of the *n*π* band of Z-azobenzene derivatives was set between 0.2 and 0.35. The feasible OD for every sample strongly depended on the intensity of the ππ* band and the resulting white light intensity around 350 nm, which was a crucial wavelength for probing. Depending on the OD of the sample, excitation energies of 45−60 nJ were used. All excitation pulses had a pulse width from 90−100 fs, and the experiments were performed under magic angle conditions to eliminate anisotropic contributions.

#### Kinetic Modeling

In addition to lifetime density analysis (OPTIMUS),^39^ global target analysis (GTA)^44,51^ was performed. In GTA, kinetic models are directly fitted to the experimental data and subsequently evaluated. This approach yields species-associated difference spectra (SADS), which encapsulate the spectral information on the chemical species involved in the model. Furthermore, kinetic rates for each reaction step, along with the corresponding lifetimes and decay-associated difference spectra (DADS), are obtained. For this analysis, a minimal four-state kinetic model was selected (Figure S10A). This model comprises the species directly following excitation, the remaining S_1_ population, the hot ground state population, and the resulting (*E*)-isomer. Consistent with the previous literature,^32^ the initial excited state primarily relaxes to the ground state (*k*_2_), with only a minor population persisting in the excited state (*k*_1_). This excited-state population subsequently relaxes to the hot ground state via *k*_3_. The hot ground state then undergoes relaxation to the (*E*)-isomer (*k*_5_) or decays (*k*_4_). An additional rate constant, *k*_6_, accounts for the constant offset observed due to the (*E*)-isomer formation. It is important to note that a significant portion of the excited population relaxes back to the initial (*Z*)-isomer, a process accounted for by the decay of the hot ground state with *k*_4_. Attempts to employ models with more than four states resulted in compensating lifetimes and physically unreasonable SADS, precluding differentiation between two distinct hot ground states (one for the (*E*)-isomer and one for the (*Z*)-isomer). It is recognized that this type of analysis is susceptible to overparametrization and nonunique solutions. Therefore, the physical reasonableness of the obtained SADS and DADS (Figure S10B and C) served as a crucial criterion for selecting the most appropriate kinetic model. The populations presented in Figure 2 were calculated based on the chosen kinetic model.

### Computational Methods

All calculations were performed in the MNDO2020 software package. These were performed by employing the OM2/MRCI method, as implemented. Within the dynamics simulations, the analytical variant of energies, gradients, and nonadiabatic coupling terms was used. The ground state was determined by employing the restricted open-shell Hartree−Fock (ROHF) method. The active space for the multireference configuration interaction (MRCI) procedure included ten orbitals. These are the four highest doubly occupied π-orbitals, the singly occupied nitrogen lone-pair and one singly occupied π-orbital, where the last two are the highest occupied molecular orbital (HOMO) and lowest unoccupied molecular orbital (LUMO) in the closed-shell case, respectively, and four unoccupied π-orbitals. With this, three reference configurations were chosen. These were the open-shell configurations as well as the two closed-shell determinants that correspond to the doubly occupied HOMO and LUMO case. The MRCI procedure included all configurations that included single and double excitations from the reference configurations. Relevant minima were optimized and subjected to frequency calculations to confirm them as minima without imaginary frequencies. This was done with and without employing the Grimme dispersion correction D3 with Becke-Johnson damping.^52^

The excited-state dynamics employed a Tully fewest switches surface hopping algorithm, employing an empirical decoherence correction^53^ of *d* = 0.1 hartree. The initial structures and velocities were obtained from Born−Oppenheimer ground state dynamics, generating a microcanonical ensemble. The dynamics runs were initiated in the first singlet excited state, and nuclear motion was followed using the velocity Verlet algorithm^54^ with a constant time step of 0.05 fs, where the electronic Schrodinger equation was propagated in 0.001 fs steps. In total, 1000 trajectories per derivative were run with and without dispersion correction. They were followed for up to 500 fs.

## Supplementary Material



## Notes

Experimental procedures and characterization data for the azobenzene derivatives (PDF)
