# Supplementary material for: Dispersion-Controlled Excited-State Dynamics in Azobenzene Photoisomerization
Source: J Am Chem Soc. 2025 Dec 30;148(1):997–1003. doi: 10.1021/jacs.5c16915 (PMC12814177; doi:10.1021/jacs.5c16915)
Supplement: Supplementary file 1 [file ja5c16915_si_001.pdf]

Supporting Information

Dispersion-controlled excited-state dynamics in Azobenzene Photoisomerization

Torben Sassmannshausen,[+,†] Nils Oberhof,[+,‡] Marcel A. Strauss,[¶] Chavdar Slavov,[#]
Hermann A. Wegner[*,¶], Andreas Dreuw[*,‡] and Josef Wachtveitl[*,†]

† Institute of Physical and Theoretical Chemistry, Goethe University, Frankfurt, 60438, Germany
‡ Interdisciplinary Center for Scientific Computing, Heidelberg University, Heidelberg, 69120, Germany
¶ Institute of Organic Chemistry, Justus Liebig University Giessen, Giessen, 35392, Germany, and Center of Material Research (LaMa/ZfM), Justus Liebig University Giessen, Giessen, 35392, Germany
# Department of Chemistry, University of South Florida, Tampa, Florida, 33620, United States
+ These authors contributed equally
** Corresponding Authors*

E-mail: wveitl@theochem.uni-frankfurt.de, dreuw@uni-heidelberg.de,
Hermann.A.Wegner@org.chemie.uni-giessen.de

Additional Figures

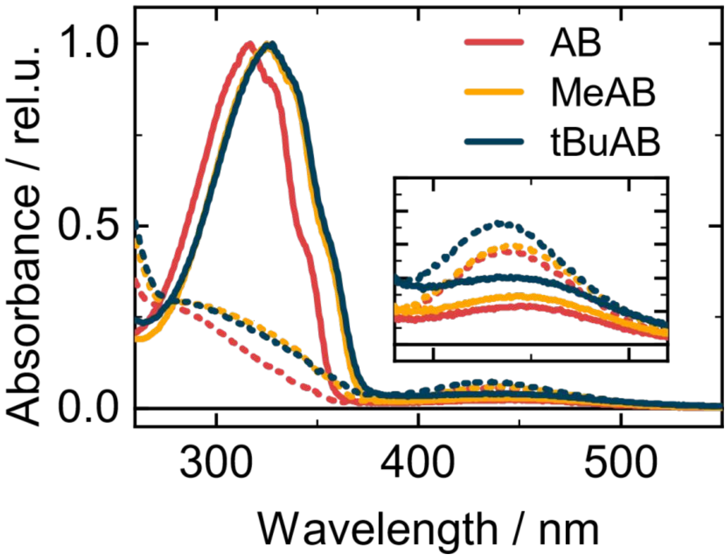

Figure 1: Absorbance spectra (solid lines) and photostationary states (dashed lines) of (*Z*)-azobenzene derivatives in octane. The photostationary states were generated by 325 nm LED illumination.

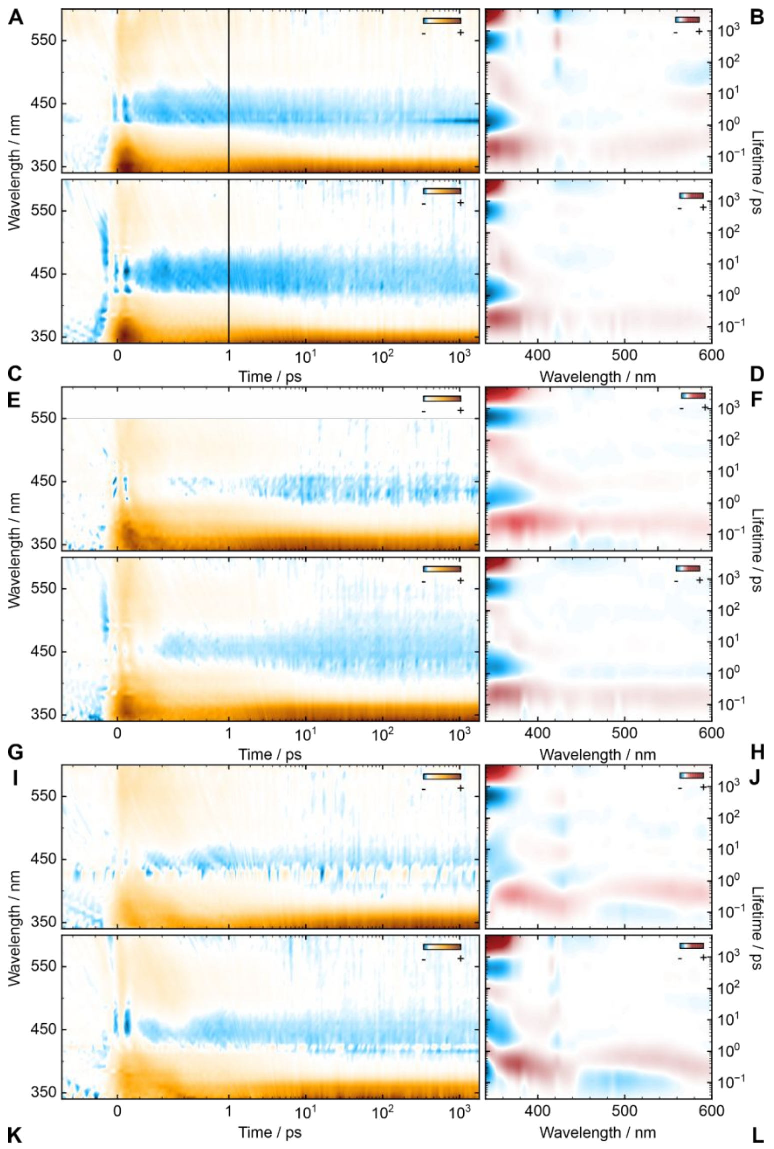

Figure 2: (A-D): The upper panel shows the ultrafast data and the respective LDM of (*Z*)-AB in MeCN while the lower part shows the same in octane. (E-H) and (I-L) show similar data for (*Z*)-MeAB and (*Z*)-*t*BuAB.

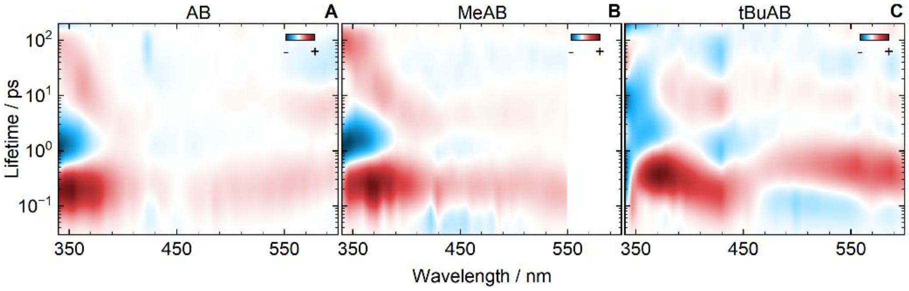

Figure 3: A-C: Lifetime density analysis of the transient absorption data in octane for (*Z*)-AB, (*Z*)-MeAB and (*Z*)-*t*BuAB respectively. The positive components describe a rising negative or a decaying positive signal. The negative components depict a decaying negative or a rising positive signal.

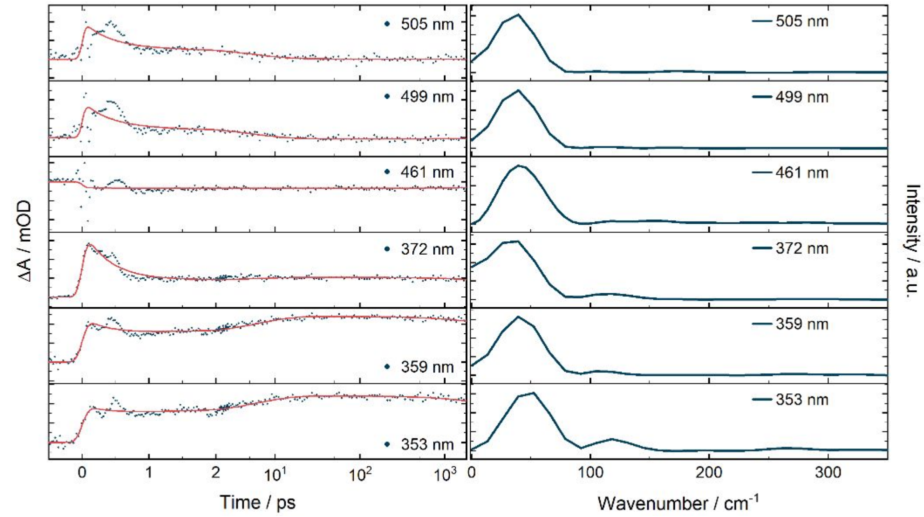

Figure 4: Single transients at selected wavelengths of (*Z*)-*t*BuAB in octane (A) and the corresponding wavenumber spectra obtained from Fourier analysis of the residuals (B).

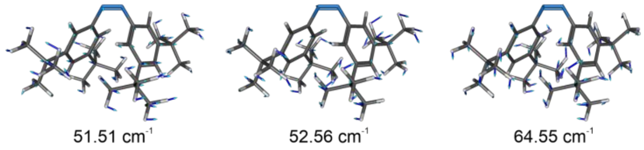

Dispersion	Mode	Characterization	Wavenumber / cm^{-1}
Corrected	1	Bending	51.51
Corrected	2	Twisting	52.56
Corrected	3	Torsion+tBu rotation	64.55
Not Corrected	1	Bending+tBu rotation	27.96
Not Corrected	2	Twisting+tBu rotation	32.34
Not Corrected	3	Torsion+tBu rotation	50.93

Figure 5: London dispersion dependent normal modes of (*Z*)-*t*BuAB with displacement vectors shown in blue. Summary of dispersion dependent normal modes of (*Z*)-*t*BuAB and the influence of the dispersion correction is shown below.

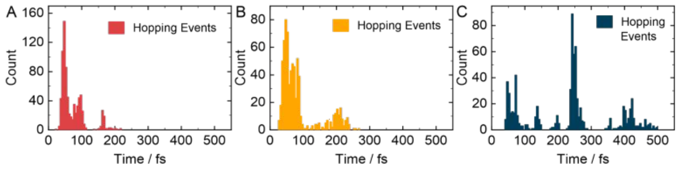

Time	(*Z*)-AB	(*Z*)-MeAB	(*Z*)-tBuAB
< 150 fs	90.9 %	83.4 %	31.2 %
> 150 fs	9.1 %	16.6 %	/
150 - 300	/	/	46.2 %
> 300 fs	/	/	22.6 %

Figure 6: Histogram of all hopping events for (*Z*)-AB (A), (*Z*)-MeAB (B) and (*Z*)-*t*BuAB (C). Distribution of the populations within the different time intervals for all three compounds below.

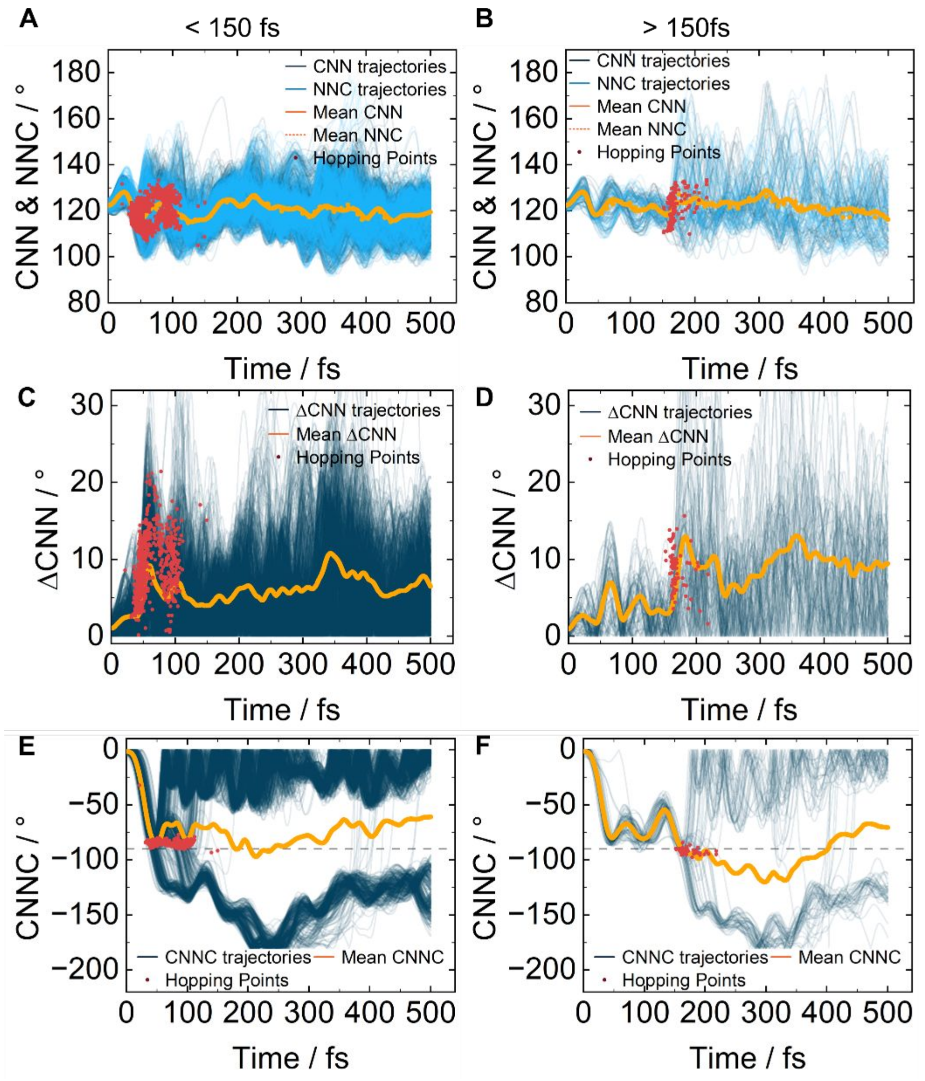

Figure 7: Time evolution of the CNN and NNC angle (A and B), the difference between the CNN and NNC angle (C and D), and of the CNNC angle (E and F) for (*Z*)-AB. The left column displays the hopping events < 150 fs, while the right shows the events > 150 fs. All hopping events are marked as red dots and the mean trajectories are colored in yellow.

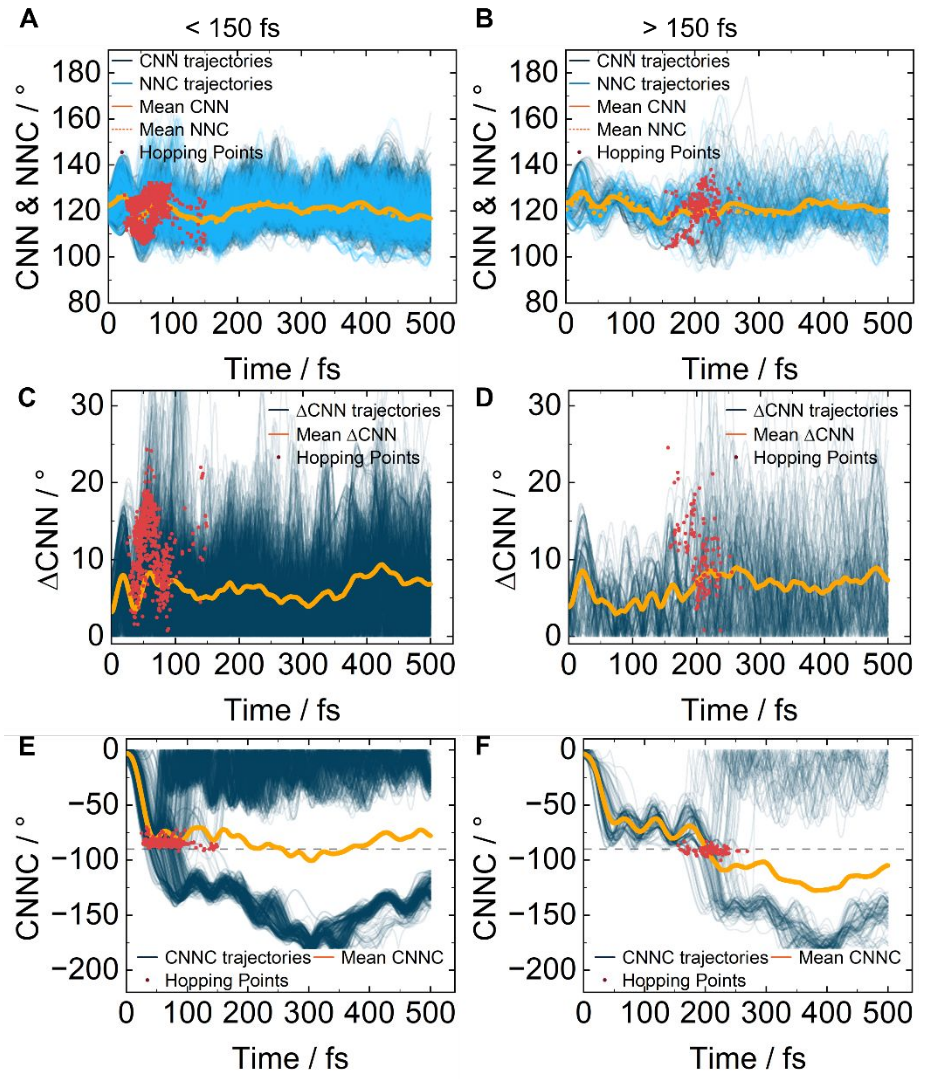

Figure 8: Time evolution of the CNN and NNC angle (A and B), the difference between the CNN and NNC angle (C and D), and of the CNNC angle (E and F) for (*Z*)-MeAB. The left column displays the hopping events < 150 fs, while the right shows the events > 150 fs. All hopping events are marked as red dots and the mean trajectories are colored in yellow.

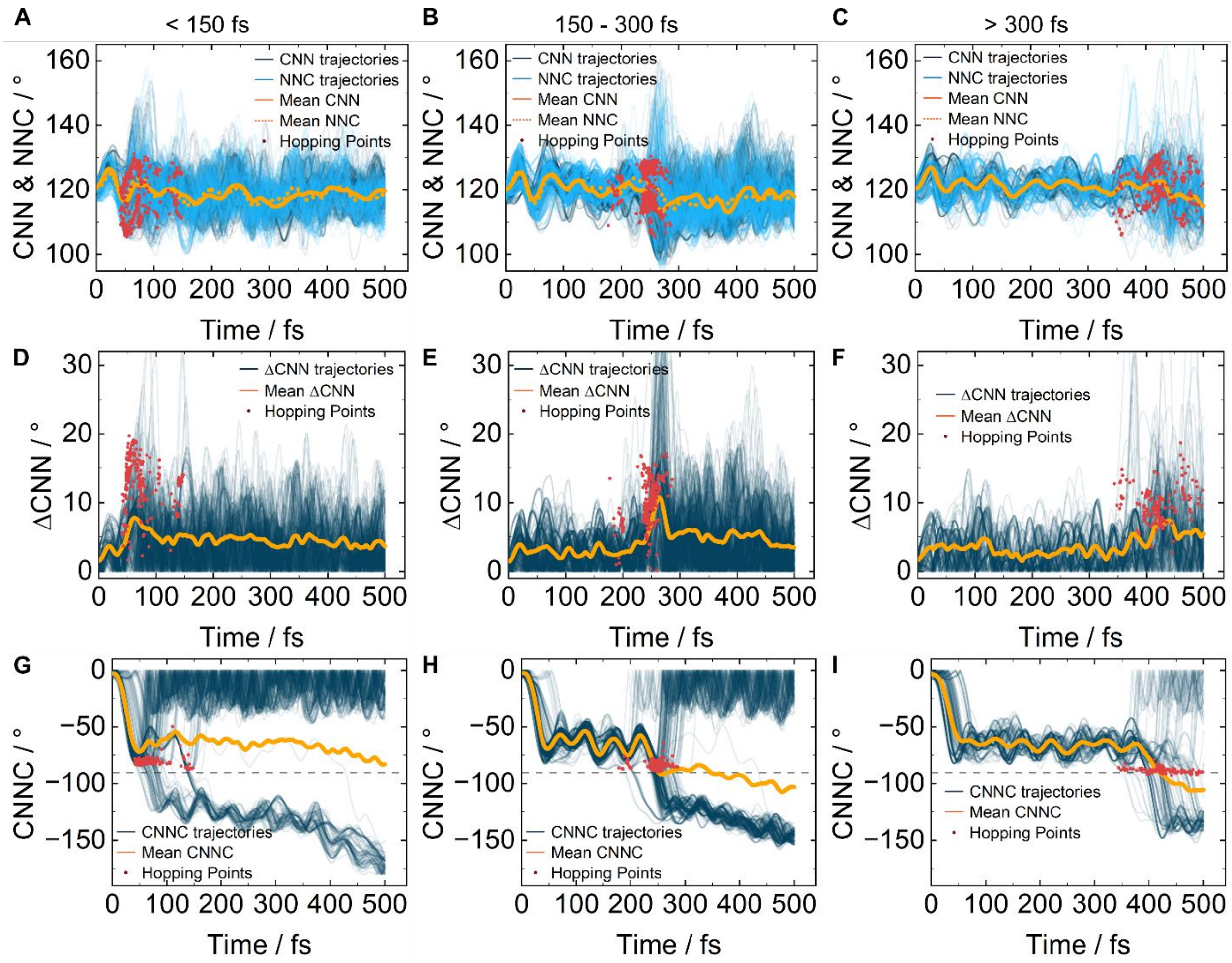

Figure 9: Time evolution of the CNN and NNC angle (A and B), the difference between the CNN and NNC angle (C and D), and of the CNNC angle (E and F) for (*Z*)-*t*BuAB. The left column displays the hopping events < 150 fs, while the right shows the events between 150 fs and 300 fs. The column on the right shows the hopping events after 300 fs. All hopping events are marked as red dots and the mean trajectories are colored in yellow.

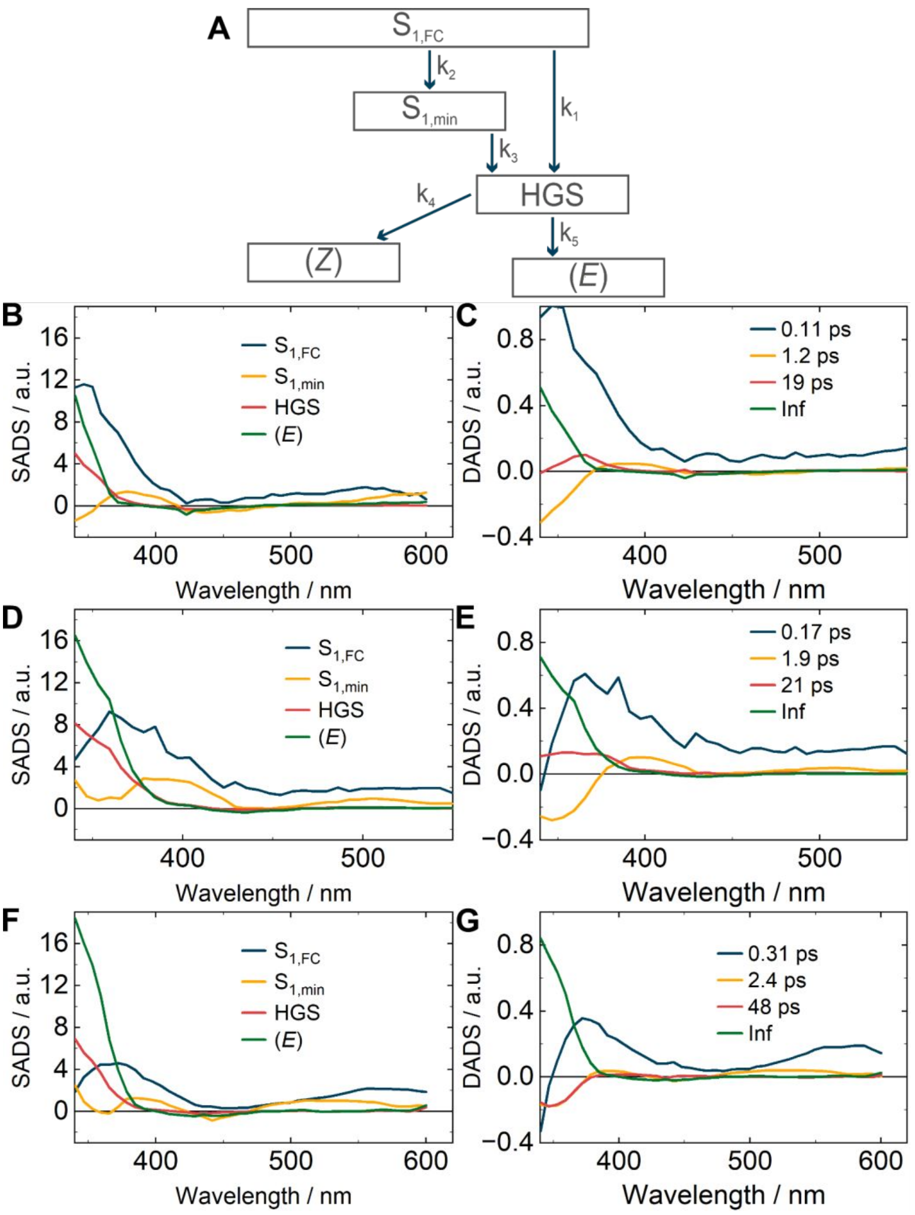

Figure 10: Kinetic model used for the global target analysis and the corresponding SADS and DADS for AB (B, C), MeAB (D, E) and *t*BuAB (F, G).

Quantum Yields

Table 1: Quantum yields for all three azobenzene derivatives in both solvents. All samples were illuminated with 420 nm and the absorbance change was detected at 440 nm.

Sample	QY (mean ± std)
AB MeCN	41% ± 2%
MeAB MeCN	38% ± 3%
*t*BuAB MeCN	37% ± 2%
AB octane	42% ± 2%
MeAB octane	39% ± 3%
*t*BuAB octane	39% ± 3%

Our determined quantum yields for AB are in range of the values known in literature.[1]

References:

(1) Bandara, H. M. D.; Burdette, S. C. Photoisomerization in Different Classes of Azobenzene. *Chemical Society Reviews* **2012**, *41* (5), 1809–1825. https://doi.org/10.1039/c1cs15179g.
